# Feature-First Add-On for Trajectory Simplification in Lifelog Applications

**DOI:** 10.3390/s20071852

**Published:** 2020-03-27

**Authors:** JunSeong Kim

**Affiliations:** School of Electrical and Electronics Engineering, Chung-Ang University, Seoul 06974, Korea; junkim@cau.ac.kr; Tel.: +82-2-820-5294

**Keywords:** lifelog, feature points, trajectory simplification, context, GPS data

## Abstract

Lifelog is a record of one’s personal experiences in daily lives. User’s location is one of the most common information for logging a human’s life. By understanding one’s spatial mobility we can figure out other pieces of context such as businesses and activities. With GPS technology we can collect accurate spatial and temporal details of a movement. However, most GPS receivers generate a huge amount of data making it difficult to process and store such data. In this paper, we develop a generic add-on algorithm, feature-first trajectory simplification, to simplify trajectory data in lifelog applications. It is based on a simple sliding window mechanism counting occurrence of certain conditions. By automatically identifying feature points such as signal lost and found, stall, and turn, the proposed scheme provides rich context more than spatio-temporal information of a trajectory. In experiments with a case study of commuting in personal vehicles, we evaluate the effectiveness of the scheme. We find the proposed scheme significantly enhances existing simplification algorithms preserving much richer context of a trajectory.

## 1. Introduction

Lifelog is a record of a person’s daily life in varying amounts of detail [1,2,3]. It represents the totality of life experience and one can potentially improve work performance or find unconscious behavior through self-tracking. Since people divide living space for a specific purpose one can easily figure out a variety of context such as businesses and activities from location data. For example, a typical house consists of living room, bedroom, bathroom, kitchen, and dining room. People sleep at bedroom, watch TV at living room, and cook at kitchen but hardly eat at bathroom. User’s location has a huge implication and is one of the most common information for logging a person’s life [3,4,5]. By understanding one’s spatial mobility we can infer the behavior and attitude of the person in various situations in everyday life. 

The availability and affordability of GPS (Global Positioning System) technology provides a simple yet powerful harness for collecting mobility data [4,5,6]. With GPS, we can capture consistent and accurate spatial and temporal details of a movement. However, most GPS receivers generate a huge amount of data making it difficult to process and store them. To overcome the difficulty, various simplification algorithms for trajectory data have been proposed. The basic idea is to discard redundant or less important data points preserving the context of the original trajectory data. Most of the existing algorithms, however, mainly focus on accuracy and storage size [7,8,9,10]. An error-bounded approach tries to minimize the number of data points in simplified trajectory while it maintains a specified approximation error ε. A size-bounded approach, on the other hand, tries to minimize the approximation error of simplified trajectory with a specified number of data points. Typical trajectory simplification is a process to balance between accuracy and storage size. In the meantime, information loss from the original trajectory is unavoidable under certain criterion. There is no one-fits-all solution and application-specific algorithms are desirable to match the type of information utilized by the applications. 

In this paper, we present a generic add-on algorithm for trajectory simplification in lifelog applications. We call it *feature-first trajectory simplification* (FFTS). Existing trajectory simplification algorithms can be classified into two types based on the applications’ mode: *Batch* (*offline*) and *online* algorithms. Batch algorithms require an entire trajectory data before doing any simplifying operations. These generally achieve a good balance between accuracy and storage size at the cost of higher computation. DP, Bellman, TD-TR are examples of a batch algorithm [10,11,12]. Online algorithms, on the other hand, work for streaming trajectory data in real-time applications. These generally cannot achieve optimal results by trying to maintain the relatively important data points within a local buffer of restricted size. STTrace, SQUISH, DOTS are examples of an online algorithm [10,13,14]. The FFTS works either by itself or with any existing simplification algorithm, which can be either a batch or an online algorithm. By identifying feature points, such as signal lost and found, stall, and turn, it preserves context more than spatio-temporal information of a trajectory. In order to identify feature points the proposed scheme utilizes additional information such as GPS status, speed, track angle, etc., which a GPS receiver naturally provides, in addition to the location and timestamp information. Since it is based on a simple sliding window mechanism its processing complexity and local storage requirements are very low. Moreover, by splitting original trajectory into segments by the feature points in advance it provides the opportunity for reducing processing time of its combined algorithm, if any. In experiments with a case study of commuting in personal vehicles we inspect the effectiveness of the scheme. The experimental results show that the FFTS preserves much richer context of a trajectory by taking feature points in simplified trajectory and that the simplification with FFTS outperforms significantly the one without FFTS at minimal cost in compression rate.

The remainder of this paper is organized as follows. In Section 2, we briefly describe trajectory data, which most GPS receivers provide by nature. In Section 3, the feature-first trajectory simplification algorithm is presented in detail. Then, Section 4 provides a comprehensive evaluation and analysis on the experimental results. Finally, Section 5 summarizes our results and conclusions. 

## 2. GPS Trajectory Data

GPS is a network of satellites and provides users with positioning, navigation, and timing services in a wide range of personal and commercial applications. GPS satellites continuously send out radio signals on their orbital information and onboard clock time. A GPS receiver on the ground calculates its own position based on the signals from the satellites. The positioning works on a simple concept of *trilateration* using the locations of orbiting GPS satellites and the distance from those satellites to the receiver on the Earth [15,16]. In order to determine the coordinates on the three-dimensional space (longitude, latitude, altitude) of the earth at least three satellites are necessary. In addition, an extra satellite is used for synchronization of clocks in use. The onboard atomic clocks of the satellites are highly accurate and are synchronized with each other. The clock at the receiver, however, is not synchronized precisely with the clocks of the satellites. As of 14 January 2020, there are 31 operational satellites in the GPS constellation ensuring that at least four satellites are visible at all time anywhere on the Earth [15]. 

Most GPS receivers provide data in the form of ready to be used in typical location-based applications, however, only when they can see at least four satellites. This is called a *fix*. A GPS receiver starts spitting out data when you turn it on even if it does not have a fix. The *time-to-first-*f*ix* (TTFF) depends on the startup mode of a receiver. A GPS receiver, in general, stores its last valid position and time information with almanac and ephemeris data to predict which satellites are in visible range. If a receiver has no such information, then it is up in *cold start* mode taking several minutes to get a fix. If a receiver has all the information, then in *hot start* mode for the quickest fix taking typically dozens of seconds. In *warm start* mode it takes longer than a hot start but not as long as a cold start. 

**Figure 1:**
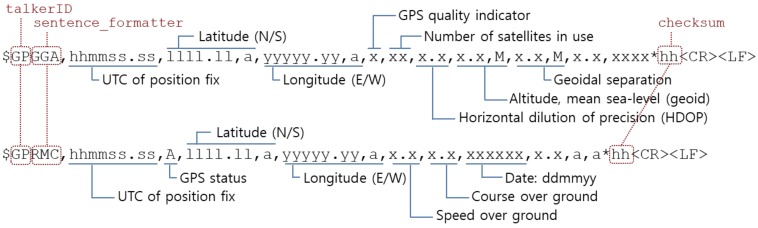
The National Marine Electronics Association (NMEA) format of GPS fix data (GPGGA) and recommended minimum specific GNSS data (GPRMC) sentences.

GPS receivers generally output information in NMEA (National Marine Electronics Association) 0183 format, which is an ASCII interface standard for marine electronic devices [17,18]. There are a few different kinds of NMEA sentence and all sentences begin with the character "$" and end with the sentence termination delimiter "<CR><LF>". Each NMEA sentence consists of an address field, a data field, and a checksum such that "$<address>, <data>*<checksum><CR><LF>". The first field of <address> consists of the *talker identifier* and the *sentence formatter*. The talker identifier indicates where the data comes from and it is "GP" for GPS. The sentence formatter specifies the number of data subfields in the sentence, the type of data they contain and the order in which the data subfields are transmitted. The data field <data> in each sentence contains the number of subfields, which is specified by the sentence formatter, separated by commas ",". Most GPS receivers provide "GPGGA" (GPS Fix Data), "GPRMC" (Recommended Minimum Specific GNSS Data), "GPGSA" (GNSS DOP and Active Satellites), "GPGSV" (GNSS Satellites in View) sentences by default. GPGGA and GPRMC are most commonly used and Figure 1 shows the format of them. You can see that there are redundant data between sentences and easily imagine the meaning of the subfields for UTC (Coordinated Universal Time) of position fix, latitude in the northern or southern hemisphere, longitude in the easterly or westerly, speed over ground in knots, course over ground in degree true. The following is a brief description of the remaining subfields:

- **GPS status**: The data set quality (V = invalid, A = valid)
- **GPS quality indicator**: The GPS fix type (0 = no GPS, 1 = GPS SPS, 2 = DGPS, 3 = GPS PPS). It indicates whether the GPS receiver has fixed onto satellites’ data and received enough data to determine the location.
- **Horizontal dilution of precision (HDOP)**: DOP tells the effect of satellite geometry on measurement accuracy. The precision of the calculated position is reduced when GPS’ four reference satellites are close together. HDOP describes the influence of satellite geometry on the position upon a 2D plane. The positional error is proportional to the value of HDOP.
- **Altitude, mean sea-level (geoid)**: The geoid is a theoretical surface, which is defined by the gravity, of the Earth. It is often used as a reference level for measuring height.
- **Geoidal separation**: The difference between the WGS-84 earth ellipsoid surface and the geoid in meter. An ellipsoid is an approximation of the true shape of the Earth for convenient manipulations.

In most literatures on GPS trajectory study, only the minimum information of longitude, latitude, and time is considered. GPS receivers, however, have a processor and an antenna that directly receive data from the satellites and compute its position on the fly. The processor on a GPS chipset is responsible for all of the calculations and user interfaces, as well as analog circuits for the antenna. Users can change the configurations of a GPS receiver for sampling frequency, sentence selection, baud rate, etc. That is, GPS receivers provide much more information than the minimum by nature and it is a waste not to use this information in trajectory study. 

## 3. The Feature-First Trajectory Simplification

We assume that the raw data stream consists of a series GPS data, denoted as *P* = {*p_t_*_0_, *p_t_*_1_, *p_t_*_2_, …, *p_tn_*, …} where *p_tn_* = (id, *x*, *y*, *t*, *d*_1_, *d*_2_, …, d*_m_*, …) is referred to as a *data point*. Each data point basically contains object ID, longitude x, latitude y, and time stamp t. It also has other information *d_m_*, which a GPS receiver provides by nature. In this study, we use the additional information of GPS quality indicator, number of satellites in use, HDOP from a GPGGA sentence and of GPS status, speed and course over ground from a GPRMC sentence, as well as UTC time and date, latitude, longitude for spatial and temporal information. The objective of the *feature-first trajectory simplification* (FFTS) is to find a subset of *P* denoted as *P′*, which represents *P*. Each data point in *P′* needs to have an additional attribute of *feature type* but may exclude extra information other than the minimum of (id, *x*, *y*, *t*) depending on the applications’ requirements. We define six different types of a feature point for the simplification:

- **LOST point (*L*)**: The location where a GPS receiver has problematic satellite signals for a period longer than a predefined time *T*_fixL_.
- **FOUND point (*F*)**: The location, followed by a LOST point, where a GPS receiver has valid satellite signals for a period longer than a predefined time *T*_fixF_.
- **STALL point (*S*)**: The location where the object stops moving and remains stationary within a predefined distance *D*_maxS_ for a period longer than a predefined time *T*_movS_.
- **GO point (*G*)**: The location, followed by a STALL point, where the object moves faster than a predefined speed *S*_minG_ for a period longer than a predefined time *T*_movG_.
- **TURN point (*T*)**: The location where the object turns larger than a predefined angle Θ_minT_.
- **eXTRA tune point (*X*)**: The location, between any consecutive feature points within a trajectory, where applications demand for recording with optional requirements. Certain parameters *X_n_* may be considered depending on the requirements.

**Figure 2:**
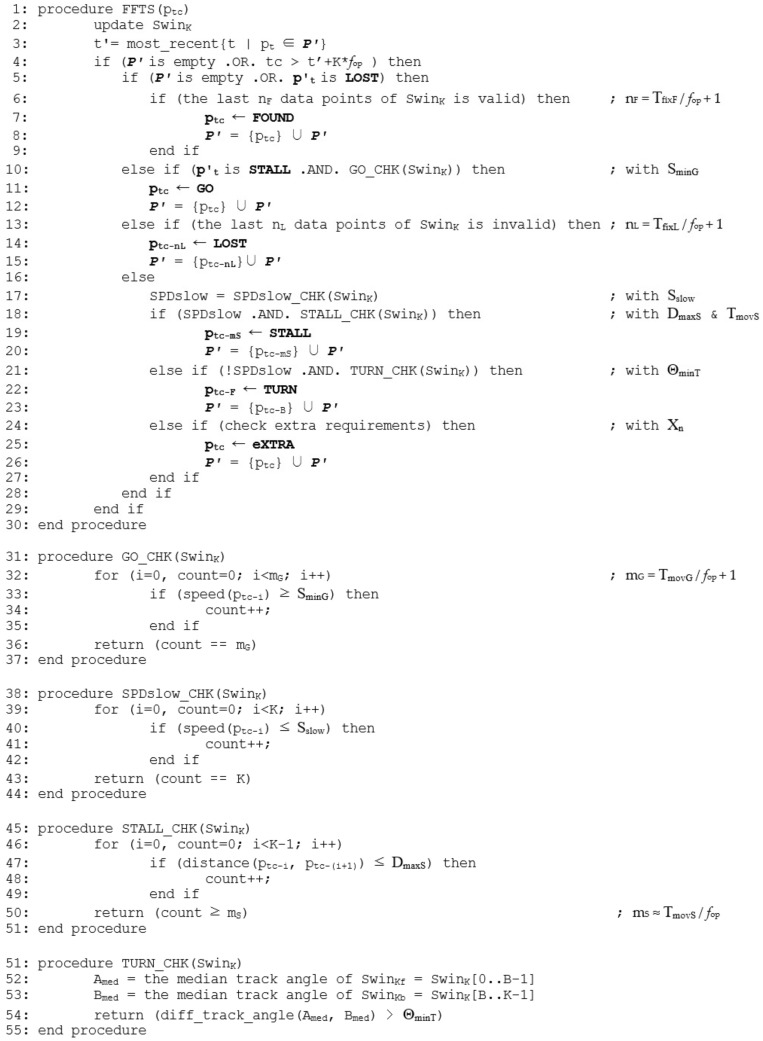
The feature-first trajectory simplification algorithm.

The parameters (*T*_fixL_, *T*_fixF_, *D*_maxS_, *T*_movS_, *S*_minG_, *T*_movG_) are set according to GPS receivers’ quality and configurations. For example, D_maxS_ is required to tolerate signal noises and errors. If the positioning signal is 100% accurate without any errors, we may set *D*_maxS_ = 0. The other parameters (Θ_minT_, *X_n_*) are set according to applications’ demands on simplification. Figure 2 shows a brief description of the FFTS algorithm. When a new data point *p_t_* is collected, FFTS tries to identify whether it belongs to one of the feature types above. If then, we add p_t_ to *P′* ⸦ *P*. Otherwise, the data point will be disregarded. To identify a feature point, it utilizes a sliding window and simply counts occurrences of certain conditions. We denote a window record that consists of the most recent *K* data point history *Swin_K_*. Let *p_tc_* be the data point at current time such that *Swin_K_* contains (*p_tc_*, *p_tc_*_−1_, *p_tc_*_−2_, …, *p_tc_*_−(*K*−1)_). We keep *Swin_K_* in two portions of front-end window Swin_Kf_ and back-end window Swin_Kb_, for our convenience. The bound between the two is parameterized by B such as *Swin_Kf_* = (*p_tc_*, *p_tc_*_−1_, …, *p_tc_*_−(*B*−1)_) and *Swin_Kb_* = (*p_tc_*_−*B*_, *p_tc_*_−(*B*+1)_, …, *p_tc_*_−(*K*−1)_). Moreover, we denote *p′_t_* the last data point added to the simplified trajectory *P′* and use the symbol "←" to assign a type to its corresponding feature point. For the type assignment of a feature point we give priority LOST/FOUND > STALL/GO > TURN > eXTRA in the order. The algorithm is straightforward and easy to see that the running time of FFTS is *O*(1) for *P′* = {*L*, *F*, *S*, *G*, *T*}. 

With a trajectory we first identify potential problematic data points. We need to be aware that GPS data are subject to various sources of errors including satellite orbit errors, satellite clock errors, receiver errors, tropospheric and ionospheric errors, multipath errors, etc. [15,16,19]. The GPS quality indicator in a GPGGA and the GPS status in a GPRMC are mainly used for inspecting the validity of a location data: Any data with status ‘V’ or fix type ‘0’ is invalid. In addition, we further refer to the number of satellites in use and the HDOP in a GPGGA. A location data with at least ‘4’ satellites and at most ‘2’ HDOP is considered reliable. Problematic data results from the loss of satellite signals such that the sky is partially or completely blocked. These include when the object is in an underground area, a tunnel, or even a valley between tall buildings. It is quite common for a GPS receiver to provide a temporarily invalid data, which is followed by valid data within a single or a couple of seconds. In order to identify points of LOST we disregard these temporarily invalid data by using the parameter *T*_fixL_. If the last *n_L_* consecutive ones among the *K* data points of *Swin_K_* are invalid, then we add *p_tc_*_−*nL*_ to *P′* with LOST status as shown at line 13–15 in Figure 2. The value of *n_L_* is determined based on GPS receiver’s sampling frequency such that *n_L_* = *T*_fixL_/*f*_op_ + 1 ≤ *K*. At the same extent, in the status of LOST, if the last *n_F_* = *T*_fixF_/*f*_op_ + 1 ≤ *K* consecutive data points of *Swin_K_* are valid, then we add *p_tc_* to *P′* with FOUND status as shown at line 5–9 in Figure 2. A FOUND point will be identified only after the corresponding LOST point is identified previously. At the beginning, when the *P′* is empty, the default status is LOST. The value of *T*_fixF_ is not necessarily the same as that of *T*_fixL_.

The operation and performance of a GPS receiver greatly depends on the acceleration of the receiver. Especially, the accuracy of location data for a stationary object is marginal and the same physical location will have different GPS coordinates from time to time [19,20]. That is, a GPS receiver does not record exactly the same location when users stay at the same place for a while. High precision would be desired but it is typical for low-cost GPS receivers and is good enough for most lifelog applications. With a valid data point we next test a range of low speed by using the speed over ground field in a GPRMC sentence. Considering the inertial nature of movement we make the decision conservatively. If the speed of all the *K* data points of Swin_K_ are slower than a predefined threshold *S*_slow_ then it is in the range of *SPDslow*. It is shown at line 17 and line 38–44 in Figure 2. In this study, a speed bound of *S*_slow_ = 15 km/h, which is determined empirically, is used. 

To identify points of STALL we consider the distance between two location data: (lat_1_, lon_1_) and (lat_2_, lon_2_). The *Haversine* formula gives the great-circle distance between two points [17]:

(1) $$d=2R\cdot \mathrm{asin}\left(\sqrt{\sin^{2}\left(\frac{{\mathrm{lat}}_{1}-{\mathrm{lat}}_{2}}{2}\right)+\cos\left({\mathrm{lat}}_{1}\right)\cdot \cos\left({\mathrm{lat}}_{2}\right)\cdot \sin^{2}\left(\frac{{\mathrm{lon}}_{1}-{\mathrm{lon}}_{2}}{2}\right)}\right),$$

where R is earth’s radius. A point of STALL occurs naturally when it is in the range of SPDslow. Calculate the distance *d* between any two consecutive data points within *Swin_K_* and, among the *K* − 1 calculations, simply count the case of *d* ≤ *D*_maxS_. If it is larger than *m_S_* ≈ *T*_movS_/*f*_op_ ≤ *K* − 1 then we add *p_tc_*_− *mS*_ to *P′* with STALL type as shown at line 18–20 and line 45–51 in Figure 2. The value of *m_S_* is somewhat related to *T*_movS_ but not exactly since in the counting we ignore the order of occurrences. It corresponds to heavy congestion or signal-related complete stops. To identify points of GO we use the speed over ground subfield in a GPRMC sentence. In the status of STALL, if *m_G_* = T_movG_/*f*_op_ + 1 ≤ *K* consecutive data points show speed higher than *S*_minG_ then we add *p_tc_* to *P′* with GO type as shown at line 10–12 and line 31–37 in Figure 2. A GO point will be identified only after the corresponding STALL point is identified previously. Ideally its location is identical to the corresponding STALL point.

To identify points of TURN we consider the course over ground in a GPRMC. As GPS receivers are concerned, it is the direction that an object is moving in and has little relationship with the direction the object is pointing to. However, a turn can be detected by calculating the difference in track angles between two data points. We take and compare the median values of the two sub-windows of *Swin_K_*: *Swin_Kf_* and *Swin_Kb_*. By taking median values in track angles we eliminate instantaneous or erroneous values. If the angle difference is larger than the threshold value Θ_minT_ then we add *p_tc_*_-*B*_ to *P′* with TURN type as shown at line 21–23 and line 51–55 in Figure 2. Note that the track angle, which is used for waypoint navigation of an object, is not accurate, especially at low speeds. Therefore, we disregard any data points in the range of *SPD*slow for the detection of TURN as shown at line 21 in Figure 2. 

Note that when a feature point is detected, a data point among (*p_tc_*_-*nL*_, *p_tc_*, *p_tc_*_−*mS*_, *p_tc_*, *p_tc_*_−*B*_) within *Swin_K_* is added to *P′* depending on the type of the feature point {*L*, *F*, *S*, *G*, *T*}. In Figure 2 it is shown at line 4 that the FFTS does not allow to have more than a single feature point within the record of window *Swin_K_*. 

As the final step, for optional requirements in the simplification we may sample extra data points between two feature points above with eXTRA type as shown at line 24–27 in Figure 2. The FFTS can collaborate with any existing trajectory simplification scheme, which is either a batch or an online algorithm, in this step. When the FFTS works by itself this final step may be skipped. In Section 4 of the case study, two well-known techniques are used as examples: *Douglas–Peucker* (DP) and *uniform sampling* (US) algorithms [10,12]. 

### 3.1. Douglas–Peucker (DP) Algorithm

It is a classic line generalization algorithm and is widely used in many geospatial applications. Initially, it takes the first and the last data points as the end points of a line segment. Next, calculate the perpendicular distance between the line segment and intermediate data points of the original trajectory. Then, add the data point with the greatest distance to the simplified one forming two new segments. Repeat the process with the new segments until the maximum distance for each line segment is less than a predefined threshold *D*_DP_. The computational complexity of DP is *O*(*n^2^*), where *n* is the number of data points within a trajectory. This scheme guarantees that the error of a discarded data point is less than the threshold and shows excellent performance in general. We use the DP as a representative batch algorithm: First, FFTS splits the original trajectory *P* into multiple small segments. Any two consecutive feature points {*L*, *F*, *S*, *G*, *T*} become the end points of each segment. Next, the DP algorithm is applied to each segment independently for the feature points of type *X*. The optional parameter *X_n_* becomes the threshold *D*_DP_ in meter. When the DP is collaborated in FFTS we denote it FFDP. 

### 3.2. Uniform Sampling (US) Algorithm

It is a simple and straightforward scheme. With a stream of data point it down-samples at fixed time intervals. Though this scheme is trivial to implement it often results in significant information loss, especially when there are drastic changes in trajectory between sampled data points. We use the US as a representative online algorithm: Assume that we sample every *i*^th^ data point using a counter. If FFTS identifies feature points {*L*, *F*, *S*, *G*, *T*} from the trajectory stream *P* it adds the data point to *P′* and resets the counter. Otherwise, it adds the *i*^th^ data point to *P′* with type *X*, when the counter is terminated, and resets the counter. The optional parameter *X_n_* becomes *T*_US_ = *i*/*f*_op_ in second. When the US is collaborated in FFTS we denote it FFUS. 

## 4. A Case Study

**Figure 3:**
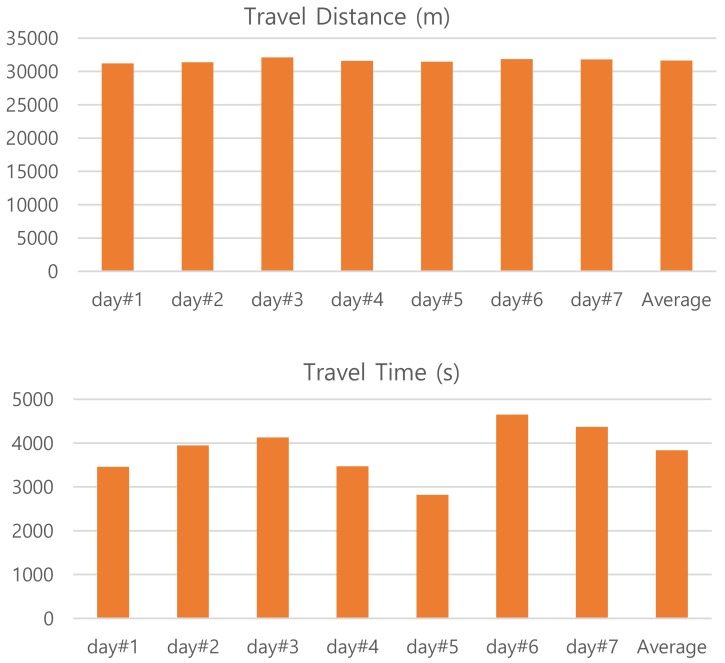
User’s commute data of seven days are summarized with respect to distance and time.

We collect user’s spatial mobility data using a GPS logger, which is built around the MediaTek’s MTK3339 chipset [21]. It can track up to 22 satellites on 66 channels in a −165 dBm sensitivity with a 15 × 15 × 2.5 mm built-in ceramic patch antenna. The GPS logger is configured to generate NMEA sentences at *f*_op_ = 1 Hz such that user’s movements are sampled at every second. A single user’s commute history by a personal automobile is traced for seven different days. Figure 3 provides a summary of each trajectory data with respect to travel distance and time. Since all the trip pass through the same route there are little differences in travel distance. One-way trip consists of around 31.6 km on average (between 31.2 and 32.1 km). Any variation in the value comes from the way of measurements, in which it accumulates point-by-point from its trajectory data. However, there are noticeable differences in elapsed time depending on traffic conditions. The travel time is 3835 s on average: The trajectory data of day#5 takes the least time (2822 s) and that of day#6 takes the most (4649 s) to travel the same commute path. 

### 4.1. Context of Trajectory by FFTS

**Figure 4:**
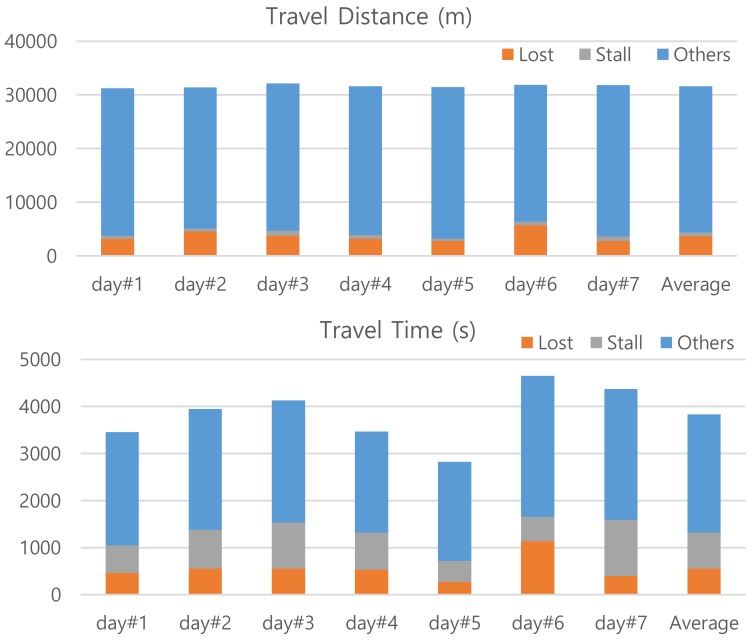
Redraw Figure 3 for more context of the trajectory data by feature types.

The context, which one might want to know in lifelog applications, of the trajectory are clearly the route and elapsed time for the trip. In addition, information on places of significance and time delay around the area within the route would be desirable. The FFTS is an effort to simplify a trajectory data without losing this context. In this experimental study, we set window size *K* = 6 so that we can simplify trajectory data with only the last 5 s history. The parameters of (*T*_fixL_, *T*_fixF_, *D*_maxS_, *T*_movS_, *S*_minG_, *T*_movG_, Θ_minT_) are set to (4 s, 2 s, 1 m, 3 s, S_slow_ = 15 km/h, 3 s, 30º). The corresponding thresholds of (n_L_, n_F_, m_S_, m_G_) for counting become (5, 3, 3, 4) considering the GPS logger’s sampling frequency of *f*_op_ = 1 Hz. Moreover, we set *B* = 3 to divide Swin_K_ into two sub-windows of Swin_Kf_ and Swin_Kb_. These values are determined empirically. Using feature points of FFTS we can redraw Figure 3 for more context of the trajectory. Figure 4 provides the same summary on travel distance and time by feature types. While the travel distance for STALL, which is ideally zero, is marginally constant the travel time for STALL varies much day-by-day. We expect the same for LOST. From the graph, however, we can see that the travel distance and time for LOST of day#6 are significantly larger than others. 

**Figure 5:**
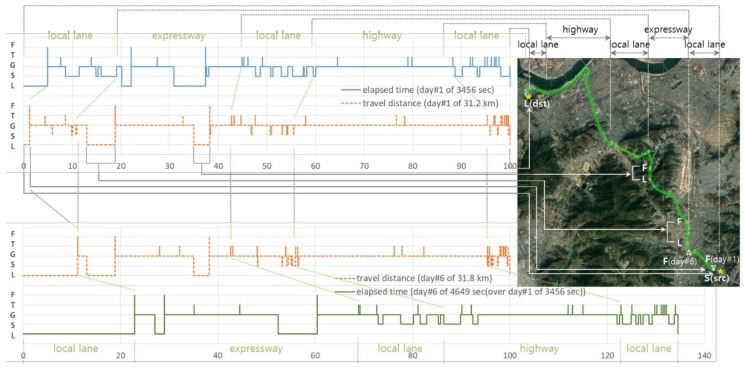
Visualization of the trajectory data of day #1 and day #6 with feature points.

In order to inspect further the trajectory data of day #6 we visualize its context with normalized travel distance and time. For comparison, the trajectory data of day#1 is drawn together. There are four graphs in Figure 5. The upper two are about the trajectory of day#1. The *y*-axis represents the type of feature points of F, T, G, S, L. The *x*-axis represents scale in % with respect to travel time of 3456 s for the top most line graph or scale in % with respect to travel distance of 31.2 km for the second dotted line graph. The lower two graphs are the same context but about day#6, which we want to examine. Note that the *x*-axis of the bottom most line graph is normalized to travel time of 3456 s of day #1 instead of 4649 s, which becomes 134.5 %, of day #6 for direct comparison between them. Figure 5 also provides the trajectory data on Google map for convenience. The commute path begins at the bottom-right point of yellow star labeled by “S (src)” and ends at the top-left point labeled by “L (dst)”. In between several dots, which represent different feature types, are shown so that we can match the graphs with the path on the map.

**Figure 6:**
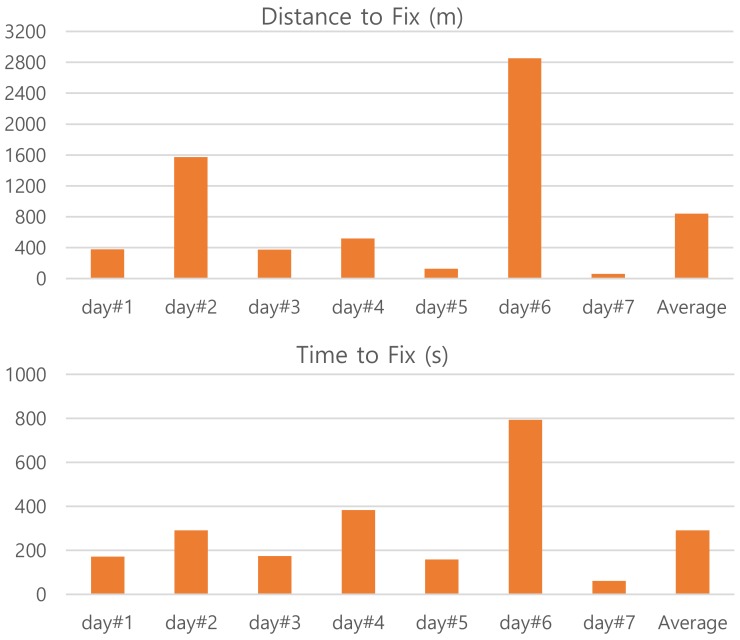
Comparison of start-up periods for the trajectory data.

From the graphs we can see three occurrences of LOST and FOUND period. The first one at the beginning is about start-up periods of the GPS receiver. The start-up time of day#6 takes much longer than that of day#1. The GPS logger is powered on at the point “S (src)”. The first FOUND points are shown on the map by a backward-pointing arrow with the label of “F(day#1)” and by a forward-pointing arrow with the label of “F (day #6)”. In Figure 6, we compare the start-up periods of the trajectory data with respect to distance and time to fix. When the GPS receiver has an estimation of current time and position it typically takes up to 3 min to acquire satellite signals. That is, the start-up of day#1 is normal and seems to be in warm start mode. On the other hand, the start-up of day #6 seems to be in cold start mode and takes more than expected. It happens from time to time and is affected by the weather condition as well. On the date of day#6 it was rain in heavy fog. The rest two LOST and FOUND periods correspond to passing tunnels. Those locations can be considered as *places of significance* for the commute path [6,22,23]. 

The occurrences of STALL or TURN might be slightly different time-by-time even on the same travel path. You may pass a traffic light without stop when it is on green and turn either gently or fiercely on a curve. However, they can be used for identifying places of significance if we have trajectory data large in volume. In addition, with the feature points of FFTS in simplification we can have much richer context of a trajectory. For example, in Figure 5 we distinguish types of road such as highway, expressway, and local lane on the commute path. We can say that, while day #6 takes more time than day #1 by 34.5 %, it spends extra time mostly on expressway and highway. These kinds of context of a trajectory data with FFTS can be used widely in trajectory data mining and lifelog applications [4,5,24]. 

### 4.2. Performance of FFTS 

In order to quantitatively compare the effectiveness of FFTS we use *perpendicular Euclidean distance* (*PED*) and *synchronized Euclidean distance* (*SED*) metrics [7,10,13]. Suppose that *p_i_* and *p_j_* are two consecutive data points of a simplified trajectory *P′*: *p_i_*_∈_ *P′* and *p_j_*_∈_ *P′*. We can consider a data point *p_k_*, which is discarded in the *P′*, of the original trajectory *P* between p_i_ and *p_j_*: $p_{k}\in P$ but $p_{k}\notin P^{\prime }$. *PED* measures the shortest distance between *p_k_* and the line segment $\bar{p_{i}p_{j}}$. In contrast, *SED* finds a virtual data point *p’_k_* on the line segment $\bar{p_{i}p_{j}}$ via interpolation. Then, it measures the distance between *p_k_* and *p’_k_*. The total error is the sum of those distances for each data point of the original trajectory *P*.

(2) $$PED=\overset{n}{\underset{k=1}{\sum }}\frac{\left|\left(y_{j}-y_{i}\right)x_{k}-\left(x_{j}-x_{i}\right)y_{k}+x_{j}y_{i}-x_{i}y_{j}\right|}{\sqrt{{\left(x_{j}-x_{i}\right)}^{2}+{\left(y_{j}-y_{i}\right)}^{2}}},$$

(3) $$SED=\overset{n}{\underset{k=1}{\sum }}\sqrt{{\left(x_{k}-{x^{\prime }}_{k}\right)}^{2}+{\left(y_{k}-{y^{\prime }}_{k}\right)}^{2}},$$

where ${x^{\prime }}_{k}=x_{i}+\frac{t_{k}-t_{i}}{t_{j}-t_{i}}\left(x_{j}-x_{i}\right)$ and ${y^{\prime }}_{k}=y_{i}+\frac{t_{k}-t_{i}}{t_{j}-t_{i}}\left(y_{j}-y_{i}\right)$.

**Figure 7:**
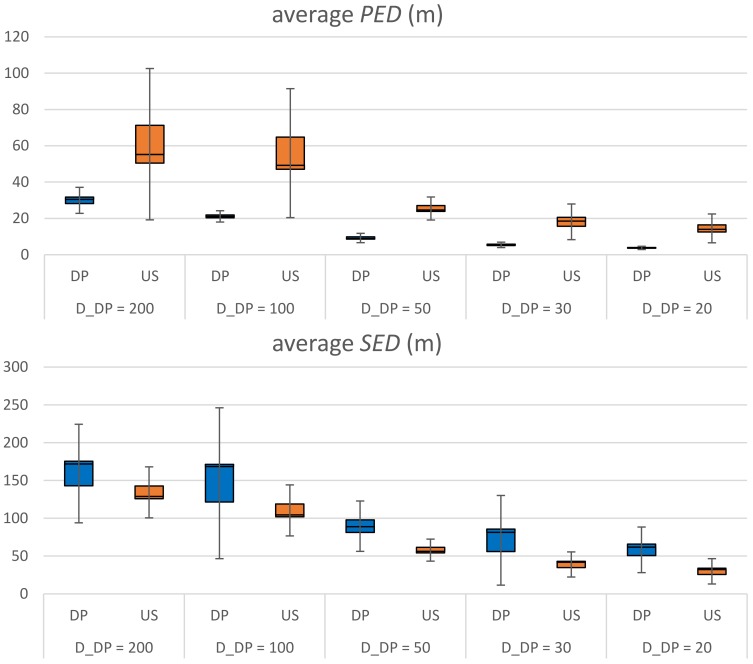
Performance comparison of Douglas–Peucker (DP) and uniform sampling (US) algorithms.

**Figure 8:**
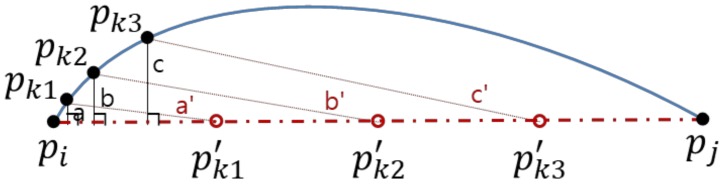
Measurements of perpendicular Euclidean distance (*PED)* and synchronized Euclidean distance (*SED)*.

Figure 7 shows the performance comparison of the original DP and US algorithms by varying the threshold *D*_DP_ = 200, 100, 50, 30, 20 m. For the graph we use average *PED* or average *SED* such that they can be independent of the travel time for the same commute path. The performance data of each trajectory is provided on Table A1 and Table A2 in Appendix A. For a fair comparison we set the parameter *T*_US_ of US by the parameter *D*_DP_ of DP such that the compression rates of them are comparable. We cannot make them exactly the same because DP is an error-bounded simplification scheme while US is a size-bounded one. The compression rate of each trajectory is provided on Table A3 in Appendix A. From the graph we can see that DP significantly outperforms US, as we expected, with respect to *PED*. However, surprisingly it is not true with respect to *SED*: US is better than DP. In many literatures *SED* is a prefer metric to *PED* since it counts on both spatial and temporal aspects of a trajectory. It seems to result from several causes. First, the DP implementation in this study relies on perpendicular distance when it adds a data point to the simplified trajectory *P′*. In *SED* calculations, however, it assumes that the object moves at constant speed between two consecutive sampled data points. For instance, Figure 8 shows five data points of trajectory *P*. Let us say that *p_i_* and *p_j_* are two sampled data points of a simplified trajectory *P′*. The three discarded data points of *p_k_*_1_, *p_k_*_2_, *p_k_*_3_ lie unevenly on the original trajectory and their corresponding virtual data points of *p’_k_*_1_, *p’_k_*_2_, *p’_k_*_3_ lie evenly on the simplified trajectory. In this scenario, *SED* = *a*’ + *b*’ + *c*’ is clearly larger than *PED* = *a* + *b* + *c*. Second, errors in trajectory simplifications tend to be enlarged as the number of missing points between two sampled data points increases. That is, in Figure 8, if there were a single discarded data point of *p_k_*_2_, instead of three, then *PED* = *b* << *a* + *b* + *c* and *SED* = *b*’ << *a*’ + *b*’ + *c*’. At the same compression rate, the number of missing points between two consecutive sampled data points varies largely in DP while it is constant in US. That is, in US the *SED* of each segment can be bounded within a certain range. However, in DP the *SED* of a segment can be amplified. Note that this case study is executed in real situations, which generate highly redundant trajectory data of which compression rates are easily higher than 95+%. There are many chances to amplify the *SED* in the simplification. 

**Figure 9:**
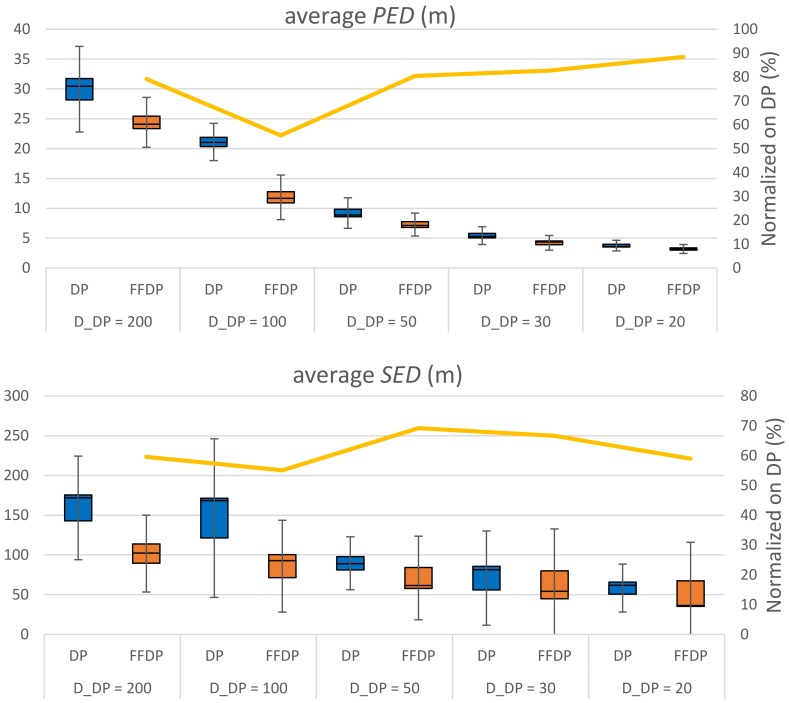
Performance comparison of DP and first-fix (FF) DP.

**Figure 10:**
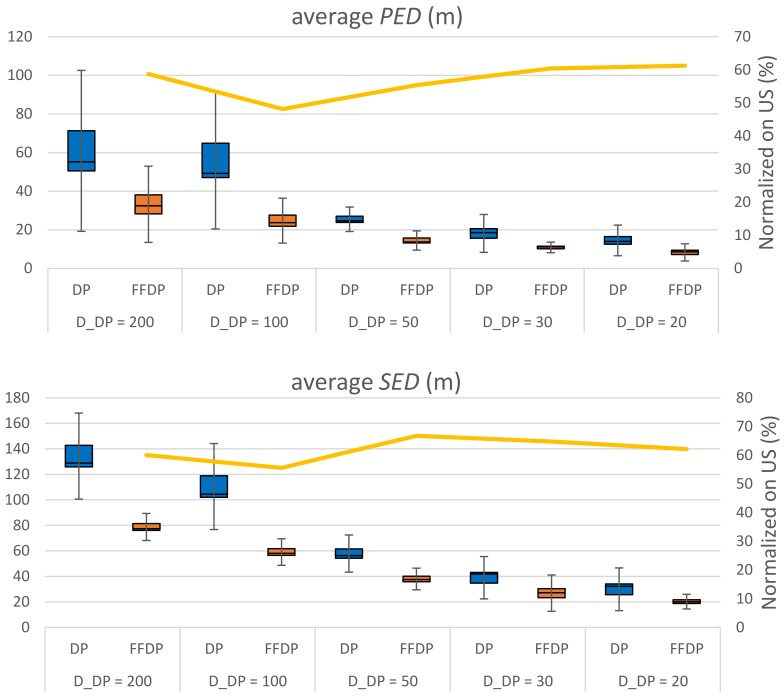
Performance comparison of US and FFUS.

**Figure 11:**
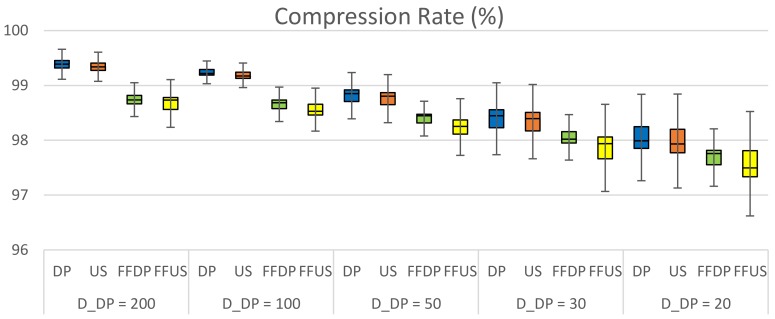
Comparison of compression rates of different simplification scheme.

Figure 9, Figure 10 show the performance comparison of DP vs. FFDP and US vs. FFUS from the same perspective, respectively. A line graph is shown together, for your convenience, on the secondary *y*-axis. It represents a normalized distance (*PED* or *SED*) of the algorithm with FFTS (FFDP or FFUS) over the original algorithm (DP or US) without FFTS. We can see that a simplification with FFTS outperforms significantly the one without FFTS. This is true for both *PED* and *SED*: For DP the reduction is 12–45 % in *PED* and 31–45 % in *SED*. For US it is 39–52 % in *PED* and 33–44 % in SED. Note that FFTS is applied first segmenting the original trajectory by feature points and that the original algorithm (DP or US) is applied next to the sub-segments for the feature type *X*. That is, the huge improvements in performance of using FFTS naturally result from taking more data points as feature points, in advance, for the simplification. Moreover, note that the number of feature points of type {*L*, *F*, *S*, *G*, *T*} for a travel path is almost constant regardless the value of the parameter *X_n_* (*D*_DP_ or *T*_US_). Figure 11 shows the compression rate of different simplification schemes in the experiments. We can see that the cost of compression rate for the performance improvements is minimal, less than 0.67 % point, by applying the FFTS. The decrease in compression rates for this kind of highly redundant trajectory data might be negligible. In addition, FFTS segmenting the original trajectory by feature points may take a benefit in processing time of the combined simplification algorithm. 

## 5. Conclusions

Lifelogging is an activity of recording user’s daily live in varying amounts of detail. User’s location is a natural form for logging a person’s life since it is of vital importance having an immense implication. Thanks to the abundant technology of GPS it becomes easier to get location data of moving objects. However, GPS trajectory data generally is huge in size making it difficult to process and store. In this paper, we presented a generic add-on algorithm, feature-first trajectory simplification (FFTS), for simplifying trajectory data in lifelog applications. It is based on a simple sliding window mechanism with additional information such as GPS status, speed, track angle, etc., which a GPS receiver provides by nature, as well as the location and timestamp. Experiments with a real trajectory data showed that the proposed scheme can preserve rich context more than spatio-temporal information of a trajectory. By identifying feature points within a trajectory such as signal lost and found, stall, and turn travel distance and time were analyzed by feature types and were visualized in normalized scale for extra context. Moreover, from the experimental results we showed that the simplification with FFTS outperforms significantly the one without FFTS at marginal cost in compression rate. 

## Appendix A

The performance data of the seven days’ trajectory is provided for better understanding of the experimental results in Section 4.2.

**Table A1:** Comparison of average *PED* of different simplification scheme.

		Day#1	Day#2	Day#3	Day#4	Day#5	Day#6	Day#7	Average	Stdev
DP	*D*_DP_ = 200	25.7	30.5	26.7	31.7	31.8	29.6	41.9	31.1	5.3
	*D*_DP_ = 100	22.1	21.7	22.3	17.1	20.8	19.9	21.0	20.7	1.8
	*D*_DP_ = 50	9.3	10.9	8.8	10.4	8.1	8.9	8.4	9.2	1.1
	*D*_DP_ = 30	6.2	5.3	6.0	5.0	3.7	5.2	5.6	5.3	0.8
	*D*_DP_ = 20	4.5	3.5	3.7	3.6	3.0	3.5	4.3	3.7	0.5
US	*D*_DP_ = 200	45.3	55.2	62.0	51.6	80.6	49.4	99.1	63.3	19.6
	*D*_DP_ = 100	45.9	48.2	49.2	72.3	57.4	37.6	94.1	57.8	19.3
	*D*_DP_ = 50	26.2	28.8	24.5	24.7	27.9	23.2	22.7	25.4	2.3
	*D*_DP_ = 30	18.6	16.5	19.0	14.8	27.0	14.8	22.2	19.0	4.4
	*D*_DP_ = 20	16.5	13.2	14.0	11.8	16.4	11.3	18.7	14.6	2.7
FFDP	*D*_DP_ = 200	31.4	22.7	25.6	24.1	25.3	24.0	21.3	24.9	3.2
	*D*_DP_ = 100	10.4	13.7	10.6	11.2	12.0	13.6	11.7	11.9	1.3
	*D*_DP_ = 50	7.7	7.1	7.1	6.5	5.9	9.1	7.8	7.3	1.0
	*D*_DP_ = 30	4.0	4.4	4.9	4.4	3.6	4.7	3.8	4.2	0.5
	*D*_DP_ = 20	2.9	3.5	3.4	3.1	2.5	3.4	3.2	3.1	0.3
FFUS	*D*_DP_ = 200	30.8	25.0	25.8	36.0	41.8	32.4	40.3	33.2	6.6
	*D*_DP_ = 100	22.5	23.7	27.1	29.1	28.2	21.2	19.4	24.5	3.7
	*D*_DP_ = 50	15.7	15.7	12.1	13.7	17.1	13.4	13.1	14.4	1.8
	*D*_DP_ = 30	11.2	11.6	9.9	9.5	11.5	10.4	11.9	10.9	0.9
	*D*_DP_ = 20	7.1	6.8	8.6	10.5	9.1	7.3	9.8	8.4	1.4

**Table A2:** Comparison of average *SED* of different simplification scheme.

		Day#1	Day#2	Day#3	Day#4	Day#5	Day#6	Day#7	Average	Stdev
DP	*D*_DP_ = 200	177.9	136.0	173.1	171.9	129.1	149.8	256.9	170.7	42.6
	*D*_DP_ = 100	174.2	124.6	168.5	168.5	110.0	118.2	203.8	152.5	35.0
	*D*_DP_ = 50	88.9	71.1	93.3	102.5	87.9	74.6	137.0	93.6	21.9
	*D*_DP_ = 30	81.4	36.6	85.5	63.8	85.9	48.3	86.3	69.7	20.5
	*D*_DP_ = 20	61.8	32.8	67.5	61.8	85.0	39.7	64.1	59.0	17.6
US	*D*_DP_ = 200	131.0	119.8	128.9	126.1	154.5	125.7	211.7	142.5	32.4
	*D*_DP_ = 100	104.5	88.5	122.8	115.0	99.8	104.2	156.0	113.0	21.9
	*D*_DP_ = 50	59.1	67.3	63.9	53.8	54.7	53.2	56.3	58.3	5.4
	*D*_DP_ = 30	42.6	37.9	41.9	31.6	43.6	28.8	47.5	39.1	6.7
	*D*_DP_ = 20	33.2	26.3	34.9	25.0	32.2	22.2	39.6	30.5	6.2
FFDP	*D*_DP_ = 200	133.4	102.4	91.6	87.7	107.4	80.2	120.2	103.3	18.8
	*D*_DP_ = 100	115.8	92.8	70.8	72.0	96.0	69.4	104.5	88.8	18.4
	*D*_DP_ = 50	106.9	45.5	58.9	56.7	88.2	61.6	80.1	71.1	21.4
	*D*_DP_ = 30	98.4	38.0	54.3	51.7	85.4	28.8	74.6	61.6	25.4
	*D*_DP_ = 20	92.6	36.2	36.4	34.3	83.0	24.0	52.1	51.2	26.4
FFUS	*D*_DP_ = 200	76.2	74.0	77.4	75.9	81.3	81.5	100.8	81.0	9.2
	*D*_DP_ = 100	58.4	64.9	67.2	55.0	58.1	55.8	57.1	59.5	4.7
	*D*_DP_ = 50	40.1	46.9	37.3	34.3	37.6	28.1	40.0	37.7	5.8
	*D*_DP_ = 30	31.3	27.1	29.5	20.5	23.3	23.2	33.9	27.0	4.9
	*D*_DP_ = 20	20.0	18.6	22.2	21.0	18.9	15.2	26.5	20.3	3.5

**Table A3:** Comparison of compression rates of different simplification scheme.

		Day#1	Day#2	Day#3	Day#4	Day#5	Day#6	Day#7	Average	Stdev
DP	*D*_DP_ = 200	99.30	99.39	99.39	99.33	99.22	99.57	99.52	99.39	0.12
	*D*_DP_ = 100	99.18	99.29	99.29	99.19	98.93	99.44	99.22	99.22	0.15
	*D*_DP_ = 50	98.69	98.98	98.86	98.73	98.43	99.11	98.85	98.81	0.22
	*D*_DP_ = 30	98.37	98.45	98.54	98.09	97.90	98.77	98.57	98.38	0.30
	*D*_DP_ = 20	97.99	97.94	98.17	97.77	97.54	98.44	98.32	98.03	0.31
US	*D*_DP_ = 200	99.27	99.34	99.34	99.28	99.14	99.52	99.47	99.34	0.13
	*D*_DP_ = 100	99.13	99.24	99.24	99.13	98.86	99.39	99.17	99.17	0.16
	*D*_DP_ = 50	98.63	98.93	98.81	98.67	98.36	99.07	98.80	98.75	0.23
	*D*_DP_ = 30	98.31	98.40	98.49	98.03	97.79	98.72	98.53	98.32	0.32
	*D*_DP_ = 20	97.93	97.86	98.12	97.68	97.47	98.40	98.28	97.96	0.33
FFDP	*D*_DP_ = 200	98.63	98.85	98.78	98.70	98.25	99.03	98.74	98.71	0.24
	*D*_DP_ = 100	98.57	98.78	98.68	98.58	98.15	98.92	98.69	98.62	0.24
	*D*_DP_ = 50	98.40	98.45	98.47	98.23	97.83	98.75	98.48	98.37	0.28
	*D*_DP_ = 30	98.02	98.01	98.22	97.88	97.43	98.38	98.09	98.01	0.30
	D_DP = 20	97.76	97.86	97.69	97.42	97.11	98.14	97.77	97.68	0.33
FFUS	*D*_DP_ = 200	98.51	98.78	98.73	98.61	98.25	98.98	98.78	98.66	0.23
	*D*_DP_ = 100	98.43	98.70	98.61	98.49	97.90	98.92	98.53	98.51	0.32
	*D*_DP_ = 50	98.05	98.42	98.32	98.17	97.68	98.62	98.25	98.22	0.30
	*D*_DP_ = 30	97.79	97.94	98.03	97.54	97.08	98.36	98.09	97.83	0.42
	*D*_DP_ = 20	97.50	97.48	97.76	97.19	96.90	98.08	97.86	97.54	0.40
